# Impact of Breastfeeding and Cosleeping on Early Childhood Caries: A Cross-Sectional Study

**DOI:** 10.3390/jcm10081561

**Published:** 2021-04-08

**Authors:** María Carrillo-Díaz, Ana Raquel Ortega-Martínez, Ana Ruiz-Guillén, Martín Romero-Maroto, María José González-Olmo

**Affiliations:** 1 Department of Nursing and Dentistry, Rey Juan Carlos University, 28922 Alcorcón, Spain; maria.carrillo@urjc.es; 2 Psychology Department, Jaén University, 23071 Jaén, Spain; arortega@ujaen.es; 3 Department of Orthodontics, Rey Juan Carlos University, 28922 Alcorcón, Spain; martin.romero@urjc.es (M.R.-M.); mariajose.gonzalez@urjc.es (M.J.G.-O.)

**Keywords:** breastfeeding, cosleeping, early childhood caries, oral health, oral hygiene

## Abstract

The type and duration of breastfeeding can be key factors in the development of early childhood caries (ECC). The association between nighttime feeding and ECC was investigated. Specifically, whether cosleeping is a potential mediator of children’s oral health was investigated, considering many of the etiological factors of caries. In this cross-sectional study, 212 children (aged 2–4 years) from Madrid (Spain) who breastfed at night were examined to assess the mean decayed/filled primary teeth (dft) index, and a questionnaire was administered to the mothers to collect data on the practice of breastfeeding and cosleeping and its duration, the number of nighttime feeding sessions, sugar content in the diet, dental hygiene habits, and age at first dental visit. The dft index was lower in the group that breastfed for less than 18 months (*p* = 0.02). In addition, there were significant differences in the dft index in the group breastfeeding for more than 18 months between those who coslept for 18 months or more and those who coslept for less than 18 months (*p* < 0.05), as well as between those who coslept for 18 months or more and those who did not cosleep (*p* < 0.01). In conclusion, breastfeeding at night from 18 months onwards is considered a risk factor for ECC.

## 1. Introduction

Mothers usually have a good understanding of tooth decay, but their understanding of how tooth decay can affect their children is limited. The close relationship between pediatricians and mothers provides an opportunity to conduct caries risk assessment by focusing on the key risk factors of dental caries [1], using, for example, the AAP (American Academy of Pediatrics) Oral Health Risk Assessment tool [2].

Early childhood caries (ECC) is defined as the presence of one or more carious lesions (cavitated or noncavitated), teeth missing due to decay, or fillings in any deciduous teeth in children under the age of 71 months [3,4]. It is now considered a global public health problem, although the prevalence of dental caries in developed [5] and developing countries has been declining [6]. Despite the decline, the global prevalence in children up to five years of age remains high (63%) [7], with prevalence rates of 44.4% in Australian children of this age [3], 25.9% in Japanese children aged 3 years [8], and 27.9% in American children aged 2–5 years old [8]. ECC can affect a child’s well-being, growth, and quality of life [4]. For these reasons, interest in detecting the possible etiological factors that contribute to its pathology continues to grow, and therefore, pediatricians should concentrate their anticipatory guidance on topics that can affect the risk of caries [1]. In recent years, the duration of exclusive breastfeeding has increased worldwide [9]. The AAP recommends breastfeeding for at least 12 months, continued subsequently as long as mutually desired by the mother and child [10], and following the World Health Organization (WHO) recommendations for nighttime demand, most mothers practice simultaneous breastfeeding and sleeping [11]. The relationship between cosleeping and oral health is important, and an understanding of this relationship could be beneficial for mothers, especially when teeth eruption begins (7 months) [12], but few studies have examined the relationship between cosleeping and oral health [12,13].

Cosleeping is a common practice in various cultures [11,14] and is defined as the practice of mother and child sharing a bed. Cosleeping involves both individuals sharing a bed, room, or physical closeness [14]. An inherent benefit of cosleeping is that it increases the duration of contact between the baby and mother, thereby increasing opportunities for breastfeeding and the duration of breastfeeding.

Breastfeeding has innumerable psychological and immunological benefits for the newborn [9,15,16,17,18]: It reduces the risk of mortality (caused by infection), offers protection against gastrointestinal and respiratory diseases, and prevents growth deficits during the first month of life. Benefits to the mother have also been observed, such as prevention of post-partum hemorrhage and reduced risk of anemia, type 2 diabetes, post-partum depression, and metabolic and cardiovascular diseases [19]. In addition, in women who practice prolonged breastfeeding, reduced risks of breast and ovarian cancers have also been observed [20]. In addition, breastfeeding presents a unique opportunity for mother–child bonding and enhances emotional well-being. The protective effects of breastfeeding increase with the duration.

Several studies have noted that breastfeeding at night, on demand, and for a prolonged period are factors involved in the etiology of caries [4,20,21]. The theory that night-time feeding increases the risk of caries is based on the fact that there is a reduction in salivary flow during the night, resulting in higher remaining lactose levels in the mouth and on dental plaque than would occur during the day, which leads to acidogenicity of the oral cavity [4,20,21]. Although immunoglobulins (IgA) in breast milk have been found to protect against the development of *Streptococcus mutans*, which leads to anterior tooth decay, repeated and prolonged exposure can lead to a reduction in plaque pH and thus, the decalcification of tooth enamel [21]. Some studies concluded that breastfeeding for more than 12, 18, or 24 months increased the risk of caries and recommended that the frequency and number of nighttime feedings be reduced [20]. Breastfeeding until one year of age is not associated with an increase in caries; it can even offer protection compared to formula feeding. However, recent studies have observed that in babies who are breastfed for more than 12 months, the risk of caries is increased. In addition, there is a direct connection between prolonged breastfeeding beyond 24 months and the severity of decay in deciduous dentition [19].

To date, there is much controversy surrounding the possible cariogenic potential of breastfeeding [19]. Studies that claim that prolonged breastfeeding increases the risk of ECC often have major methodological shortcomings and do not take into account the multiple factors related to the pathogenesis of this disease, such as a cariogenic diet, hygiene, and time and number of visits to the dentist. In addition, there is no research on the contribution of the practice of nighttime feeding to the oral health of the infant while considering all of the factors mentioned above. Therefore, the purpose of this study was to analyze this association. Specifically, whether cosleeping is a potential mediator of infant oral health was investigated. This study hypothesized that the practice of cosleeping would increase the risk of caries after 18 months because oral hygiene is not performed after nighttime feedings, as most of the time, the baby feeds without the mother being aware and because the number of nighttime feedings is much higher than that in the group of non-cosleepers.

## 2. Materials and Methods

### 2.1. Study Design and Setting

A cross-sectional study was carried out in February 2020. Data were collected from school visits planned by members of the Rey Juan Carlos University research team to provide free dental examinations to the participants with no additional compensation. During their visit, education was provided to the mothers about oral health habits in childhood as well as free dental check-ups for their children. The data were collected by means of a questionnaire that the mothers completed upon arrival at the clinic. The instructions for completion were provided by a member of the research team.

All 212 children (90 boys and 122 girls) where from three state schools in the southern area of the Community of Madrid (Spain). Their average age was 3.01 years (SD = 0.81), with a range of 2 to 4 years. The participants belonged to a varied sociodemographic stratum, with representation of all socioeconomic levels.

Initially, 457 mother–child pairs were screened for eligibility. The inclusion criteria were as follows: children aged 2–4 years who had been breastfed at night, and children born at term to ensure a healthy sample selection. Children with any local/systemic conditions that could affect their oral health status and children with missing/malformed/supernumerary teeth were excluded. Mothers who refused to participate in the study, mothers with mental or physical disabilities who were unable to answer the questions, and mothers who submitted incomplete questionnaires were excluded.

Mother–child dyads who reported bed-sharing every night were included in the cosleeping group to avoid the effects of confounding variables on mixed bed-sharing behavior. The non-cosleeping group included mother–child dyads that did not share a bed (excluding at exceptional times). No other inclusion/exclusion criteria were used. 

The sample size calculation was based on caries prevalence results from this region [22] with a caries prevalence for the primary dentition of 35.9%. A random sample of 212 individuals was estimated to be a representative number for the 3-year cohort, with a confidence level of 95% and based on a ±4% degree of precision. The value of around 212 individuals would be optimal if it were a randomized study, but as the sample is a convenience sample, this value was taken as a reference. Finally, 212 subject pairs were selected, including 112 (53.3%) pairs who practiced cosleeping and 100 (46.7%) pairs who did not. Seventy-seven percent of children were exclusively breastfed, and 23% were both breast and formula fed.

Informed consent was obtained from all mothers involved in the study. Ethical approval for this study was obtained from the Ethics Commission for Research Issues at Rey Juan Carlos University (2409201913019).

### 2.2. Measures

Prior to the study, the examiner was trained and calibrated by a specialist in pediatric dentistry. The result of the kappa statistics during the calibration process was 0.92 (good agreement).

The main variables were the practice of cosleeping and its duration, number of nighttime feeding sessions, sugar content in the diet, dental hygiene habits, age at first dental visit, and decayed/filled primary teeth (dft) index. They were measured as explained below.

A dichotomous question (yes/no) was used to ask mothers whether they had coslept with their child and up to what age. In addition, data were collected on the degree of drowsiness suffered by the mother during nighttime breastfeeding sessions, with 3 possible answers: conscious, sometimes conscious, or unconscious.

With regard to nighttime breastfeeding, it was asked how long nighttime breastfeeding lasted in months and the number of nighttime sessions at 12 months and at 18 months.

With regard to diet, data on added and free sugar daily intake (from rarely or never to 3 times or more per day) were collected.

To measure oral hygiene, it was asked whether oral hygiene was performed after nighttime feeding sessions (yes or no) and at what age they incorporated a toothbrush (in months).

The age at initial dental visits (in months) was also asked about. 

The children’s oral health was assessed during clinical examinations by a single pediatric dentist using a flat surface mirror, probe, gauze, and compressed air under artificial illumination (head lamp). The dft index proposed by the WHO was calculated for the number of decayed and filled teeth in the deciduous dentition. 

A pilot survey was conducted with 5 mothers who subsequently participated in the study. A researcher asked them how clear the instructions were and which questions were difficult to understand or answer. Some of the questions were rephrased for better understanding by the participants to avoid possible misunderstanding biases. 

More detailed information about the questionnaire used is described in Supplementary Table S1.

### 2.3. Statistical Analysis

The statistical analyses were performed using SPSS version 24 (SPSS Inc., Chicago, IL, USA). There were no missing values because only participants who filled out the questionnaire completely were included in the sample. Data analyses included descriptive statistics and the Kolmogorov–Smirnov test to evaluate the assumption of normality, which was confirmed. To determine possible differences, chi-square tests, ANOVA, and t-tests were performed. Scheffe (if equal variance was assumed) and Games–Howell (if not) post hoc tests were performed. The relationships between variables were analyzed using Pearson correlations. Moreover, a regression analysis was performed to determine which factors were predictors of the dft index. Significance levels were established at 0.05. 

## 3. Results

### 3.1. Sociodemographic Characteristics

The mean age (±SD) of the mothers was 32.06 (±6.4) years, whereas that of the children was 3.04 (±0.8) years. Most of the mothers (74.5%) had secondary or higher education, most were employed (64.6%), and more than half (62.7%) reported a medium socioeconomic level (Table 1). The mean age and SD at brushing initiation was 14.3 (±7.3) months, and the mean age at the first visit to the dentist was 24.6 (±10.63) months.

**Figure 1:**
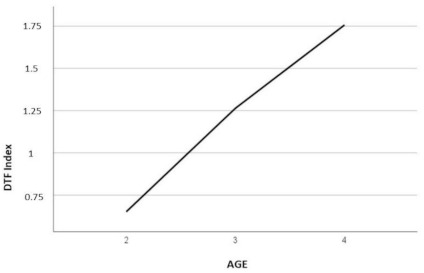
Decayed/filled primary teeth (dft) index and age.

As shown in Figure 1, the dft index increased progressively with the age of the subjects. ANOVA indicated the existence of statistically significant differences in the dft index according to the age of the children (F (2.209) = 8.21 *p* < 0.01). Specifically, differences were noted between those aged two years (M = 0.65) and four years (M = 1.75), but there were no differences between these two groups and the three-year-old group (M = 1).

### 3.2. Duration of Nocturnal Breastfeeding and Duration of Breastfeeding on the Dft Index

The subjects were divided according to the duration of lactation (<18 months and ≥18 months). We have provided the questions of the questionnaire in a table as an online appendix, adding the distribution of answers for each question. The results of the analysis show that there was a statistically significant difference between the two groups (t = 2.21, *p* = 0.028), with the dft index being lower in the <18 months of lactation group (M = 1.01) than in ≥18 months of lactation group (M = 1.53)

In the <18 months group, no significant differences were found in the dft index values on the basis of the duration of cosleeping. However, in the breastfed for ≥18 months group, there were significant differences in the dft index between those who coslept for ≥18 months (M = 2.59) and those who coslept for <18 months (M = 1.17, *p* < 0.05) as well as those who coslept for ≥18 months (M = 2.59) and those who did not cosleep (M = 1.05) (*p* < 0.01).

**Figure 2:**
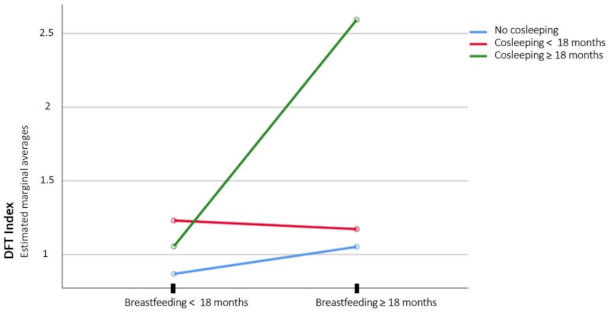
Interaction of the duration of cosleeping and duration of breastfeeding on the dft index.

The interaction of the duration of cosleeping and duration of breastfeeding on the dft index was significant (F (2.206) = 3.63; *p* = 0.028; η2 = 0.034) (Figure 2).

### 3.3. Number of Feeding Sessions at 18 Months and Breastfeeding

There were significant differences in the number of nighttime feeding sessions between those who coslept for <18 months (M = 2.27) and those who coslept for ≥18 months (M = 3.22, *p* < 0.05) and between those who coslept for ≥18 months (M = 3.22) and those who did not cosleep (M = 3.22, *p* < 0.01).

No significant differences were found in the age at first visit to the dental clinic (no cosleeping (M = 25.21 ± 9.92), cosleeping <18 months (M = 24 ± 10.97), and cosleeping ≥18 months (M = 24.26 ± 11.29); *p* = 0.74), and breastfeeding <18 months (M = 25.72 ± 11.01) and breast-feeding ≥18 months (M = 23.23 ± 9.76; *p* = 0.87); in sugar intake (0 = rarely or never, 1 = once a day, 2 = twice a day, 3 = more than twice a day, no cosleeping (M = 1.01 ± 0.74), cosleeping <18 months (M = 1.07 ± 0.96), and cosleeping ≥18 months (M = 0.82 ± 0.74); *p* = 0.27) and breastfeeding <18 months (M = 1.08 ± 0.84; *p* = 0.87 ± 0.79) and breastfeeding ≥18 months (M = 0.87 ± 0.79; *p* = 0.063); in the use of fluoride, no cosleeping (fluor N = 74, no fluor N = 25), cosleeping <18 months (fluor N = 51, no fluor N = 17), and cosleeping ≥18 months (fluor N = 34, no fluor N = 11), *p* = 0.98), and breastfeeding <18 months (fluor N = 88, no fluor N = 30) and breastfeeding ≥18 months (fluor N = 71, no fluor N = 23); *p* = 0.87); or in age at the initiation of brushing teeth, no cosleeping (M = 15.15 ± 9.24), cosleeping <18 months (M = 14.11 ± 6.37), and cosleeping ≥18 months (M = 15.20 ± 6.95); *p* = 0.66), and breastfeeding <18 months (M = 15.61 ± 8.81) and breastfeeding ≥18 months (M = 13.85 ± 6.58; *p* = 0.109).

The degree of awareness was related to the practice of cosleeping (X2(2) = 18.18, *p* < 0.01). The practice of cosleeping was associated with a lower degree of awareness than non-cosleeping.

### 3.4. Dft Index Predictor Variables

To determine whether the number of nighttime feeding sessions at 18 months was a factor associated with the number of cavities in childhood, regression analysis was performed, stratifying subjects according to whether they received nighttime dental hygiene or not. A number of feeding sessions greater than two at the age of 18 months was an independent variable, with the dft index as the criterion. The results of this analysis showed that in children who did not receive oral hygiene at night, this factor significantly predicted 57.3% of the dft index (Figure 1). However, for the subjects who received oral hygiene at night, the association was not significant (Table 2).

## 4. Discussion

The results provide empirical support for the hypotheses formulated in this study. They emphasize the possible adverse effects of the practice of cosleeping combined with nighttime breastfeeding on the oral health of infants from the age of 18 months onwards. The practice of cosleeping and breastfeeding becomes potentially aversive from this age onwards, increasing the risk of caries. However, babies who continued to breastfeed for 18 months or more but slept independently had a much lower rate of tooth decay than those who coslept.

These results can be explained by the regression model, which showed that infant oral hygiene is key in preventing early-onset caries. The mothers reported that during nighttime breastfeeding, they were sleepy and in a semiconscious state, and their babies breastfed at night very frequently, without the possibility of oral cleaning after swallowing. 

The results found in the present study contrast with previous studies on type and duration of breastfeeding and caries, such as that carried out by Iida el al. [23], who found no association between breastfeeding and its duration with caries in children, as well as that carried out by Rosenblatt et al. [24], who also found no association between the type of breastfeeding and caries. However, they did not take into account cosleeping. In more recent studies, such as those carried out by Axelsen et al. [13] and Wert et al. [12], cosleeping was considered, coinciding with the results found in the present study in terms of a higher prevalence of caries when cosleeping takes place. Nevertheless, Axelsen et al. [13] did not take into account the duration of breastfeeding and only analyzed the incidence of caries between groups of children who co-slept and those who did not. Similarly, Wert et al. [12] did not measure caries but found that breastfeeding tends to be more frequent throughout the night with cosleeping, hypothesizing that increased exposure to breast milk may increase the risk of caries. A study carried out by Peres et al. [10] also concluded that prolonged breastfeeding may be associated with nocturnal breastfeeding on demand; however, with regard to the duration of breastfeeding, they considered that the risk of developing ECC appears when breastfeeding is prolonged for 24 months or more, in contrast to the results of the present study of 18 months or more.

This study analyzed the roles played by a set of variables, such as the practice of cosleeping, breastfeeding, hygiene, diet, and visits to the pediatric dentist, but these represent only some of the many factors that could affect the development of carious lesions. Other confounding factors can be genetics, vertical bacterial transmission, drug use, and chronic disease [25,26], which were not analyzed. These variables should be taken into account when analyzing the results, but agreeing with Axelsen et al. [13], although there are more factors for the development of caries, cosleeping for 18 months or more should be considered a risk factor that increases the likelihood of a higher incidence of caries in children.

As other authors have previously proposed, it could be helpful for pediatricians to explain the results of this research to mothers in terms of breastfeeding in order to provide anticipatory guidance to families and thus, prevent caries in their children [1,10].

This research helps shed light on the poor pediatric oral health education that parents receive, since the average age at which children first visited a pediatric dentist was 24 months, a conclusion also reached by Wert et al. [12]. This figure was lower in this study than that reported in previous research, which was approximately 3.79 years (+/−1.82 years) [5]. This is because the sample of the present study attended the dentist due to an agreement between the research university team and schools to establish a preventive oral health awareness program targeting the pediatric population. The development of ECC affects the health of children and their quality of life, possibly alters development and growth, and promotes long-term problems such as malocclusions [4]. This is why the first visit to the pediatric dentist should be completed within the first year of life, or at the latest between 18 and 24 months of age, so that personalized recommendations can be provided for the prevention of ECC [4,19,27].

This research has some relevant implications for healthcare professionals. All healthcare professionals, including dentists, have a responsibility to protect and promote breastfeeding by supporting WHO recommendations and providing correct and up-to-date information based on scientific evidence. Because of the many benefits of breastfeeding, the recommendation is not to wean children early. To reduce early-onset caries, efforts should be directed at education [10]. Establishing collaborative relationships between physicians and dentists at the community level is essential for increasing access to dental care for all children and improving their oral and overall health [1].

Maternal education about prevention encompasses four fundamental aspects: first check-up of the baby by the pediatric dentist, diet, hygiene, and bacterial transmission. The importance of going to the pediatric dentist at the first dental eruption or no later than the baby’s first birthday if there is no previous pathology should be stressed. With regard to hygiene, it has recently been demonstrated that bacteria (*Streptococcus mutans*), which is responsible for caries, are present in the oral mucous membranes, even before the first tooth erupts [19]. Therefore, the need to remove the remains of milk with a silicone thimble or a damp gauze before tooth eruption should be emphasized to prevent the proliferation of bacteria and fungi (*Candida albicans*) [4]. With the eruption of the first tooth, the incorporation of an age-appropriate brush with a small amount of 1000 ppm fluoride toothpaste (similar to a grain of rice) is recommended [2,28]. With regard to diet, it is recommended to avoid added and free sugars in the diet of infants and young children (Spanish Association of Paediatrics 2020). Vertical bacterial transmission occurs when the mother exchange saliva with the child, i.e., by sharing cutlery, which is considered an inappropriate feeding practice; cleaning a pacifier with the parents’ saliva; or even sharing a toothbrush [4,19,29]. 

In summary, maternal oral health perceptions will influence the oral health behavior of their children [30].

Thus, the interaction among the triad of early-onset caries, breastfeeding, and cavities remains a pending issue for future research with a longitudinal design integrating all of the etiological factors of early-onset caries.

### Limitations

The contributions of this study should also be considered in light of its limitations. First, a convenience sample was used, which was obtained from a specific segment of the pediatric population in the Community of Madrid, potentially limiting the possibility of generalizing the results. A possible second limitation is the use of self-reported measures, which may be affected by recall bias and responses based on social desirability. It is possible that mothers’ recalls of breastfeeding, hygiene, and diet may be incomplete or inaccurate. Third, the diagnosis of caries without interproximal radiographs may also be biased towards false negative results, although many of the infants had diastemata, partly justifying the need to not perform this test. Finally, since many mothers reported being sleepy or in a semiconscious state during nighttime feedings, it is not possible to be sure that the numbers of nighttime feeding sessions were accurate; in addition to this, the retrospective measure could lead to a memory bias. Finally, the associations must be interpreted according to the observational nature of the study design, which does not allow for inferences of causality.

## 5. Conclusions

In conclusion, by analyzing the role of the different factors that contribute to the development of carious lesions, the present study helped to clarify that in children who breastfeed at night, breastfeeding from 18 months onwards is considered a risk factor for ECC because babies breastfeed frequently without waking their mother. Thus, the mother is unable to carry out oral cleaning after feedings, thereby allowing the development of carious lesions.

## Figures and Tables

**Table 1:** Sociodemographic characteristics of the sample (*n* = 212).

Variables	Frequency	Percentage (%)
**Child’s sex**		
Male	90	42.5
Female	122	57.5
**Child’s age (months)**		
2 years	66	31.1
3 years	72	34
4 years	74	34.9
**Age of mother (in years)**		
≤30	74	34.9
>31	138	65.1
**Educational level of mother**		
Low	54	25.5
High	158	74.5
**Mother’s employment**		
Employed	137	64.6
Unemployed	75	35.4
**Socioeconomic status**		
Low	14	6.6
Low-medium	18	8.5
Medium	133	62.7
Medium-High	31	14.6
High	16	7.5

**Table 2:** Prediction of the dft index from the number of nighttime feeding sessions at 18 months (>2) for the subjects who received oral hygiene at night and those who did not receive oral hygiene at night.

		R^2^	R^2^ Adjusted	F	β	t	Sign.
ORAL HYGIENE AT NIGHT	DV: dft						
YES	Number of nighttime feeding sessions at 18 months > 2	0.339	0.266	4.61	0.53	2.14	0.272
NO	Number of nighttime feeding sessions at 18 months > 2	0.591	0.573	31.83	1.57	5.64	0.000

Note: DV = dependent variable; dft = decayed/filled primary teeth index; Sign. = significance.

## Data Availability

The data that support the findings of this study are available on request from the corresponding author. The data are not publicly available due to privacy and ethical restrictions.

## Supplementary Materials

The following are available online at https://www.mdpi.com/article/10.3390/jcm10081561/s1, Table S1: Questionnaire.
