# Supplementary material for: Impact of Breastfeeding and Cosleeping on Early Childhood Caries: A Cross-Sectional Study
Source: J Clin Med. 2021 Apr 8;10(8):1561. doi: 10.3390/jcm10081561 (PMC8067957; doi:10.3390/jcm10081561)
Supplement: Supplementary file 1 [file jcm-10-01561-s001.pdf]

Supplementary Materials:

Table S1. Questionnaire.

Did you cosleep?		
Yes		112 (53.3%)
	How long did you cosleep?	
	3 months	4 (1.9%)
	6 months	32 (15.1%)
	10 months	1 (0.5%)
	12 months	29 (13.7%)
	13 months	1 (0.5%)
	15 months	1 (0.5%)
	16 months	1 (0.5%)
	18 months	19 (9%)
	24 months	16 (7.5%)
	36 months	9 (4.2%).
No		100 (46.7%)
Which type of breastfeeding did you do?		
Exclusive breastfeeding		163 (76.9%)
Mixed breastfeeding		49 (23.1%)
How long did you do night-time breastfeeding?		
3 months		2 (0.9%)
5 months		9 (4.2%)
6 months		13 (6.1%)
9 months		19 (9.0%)
12 months		13 (6.1%)
13 months		10 (4.7%)
14 months		9 (4.2%)
15 months		19 (9.0%)
16 months		7 (3.3%)
17 months		12 (5.7%)
18 months		10 (4.7%)
19 months		18 (8.5%)
20 months		21 (9.9%)
21 months		10 (4.7%)
22 months		19 (9.0%)
24 months		7 (3.3%)
25 months		8 (3.8%)
30 months		6 (2.8%)
How many times did the baby feed at an age of 12 months during the night?		
0 feedings		7 (3.3%)
1 feeding		73 (34.4%)
2 feedings		76 (35.8%)
3 feedings		49 (23.1%)
4 feedings		7 (3.3%)
How many times did the baby feed at an age of 18 months during the night?		
0 feedings		119 (56.1%)
1 feeding		36 (17%)

2 feedings<br>3 feedings<br>4 feedings<br>5 feedings<br>6 feedings	22 (10.4%)<br>19 (9%)<br>7 (3.3%)<br>7 (3.3%)<br>2 (0.9%)
Were you conscious most of the time when your child was breastfeeding during the night?	
Conscious<br>Sometimes conscious<br>Unconscious	122 (57.5%)<br>77 (36.3%)<br>13 (6.1%)
Does your child consume sugar on a daily basis, including sweets, jams, marmalades, soft drinks, fruit juices, cakes, and other sweets (such as pastries, honey, and sweetened or flavored yogurts, among others)?	
Rarely or never<br>Once a day<br>Twice a day<br>Three or more times a day	68 (32.1%)<br>83 (39.2%)<br>56 (26.4%)<br>5 (2.4%)
When did you start brushing your child's teeth?	
From 6 months of age<br>From 12 months of age<br>From 24 months of age<br>From 36 months of age	48 (22.6%)<br>99 (46.7%)<br>56 (26.4%)<br>9 (4.2%)
Does your child use fluoride toothpaste?	
Yes<br>No	159 75%<br>53 25%
When did your child go to the dentist for the first time?	
At 12 months of age<br>At 24 months of age<br>At 36 months of age<br>At 48 months of age	58 (27.4%)<br>103 (48.6%)<br>33 (15.6%)<br>18 (8.5%)
Did you perform oral hygiene after each night-time feeding?	
Yes<br>No	63 (29.7%)<br>149 (70.3%)
